# Evaluating the treatment effectiveness and efficiency of Carriere Distalizer: a cephalometric and study model comparison of Class II appliances

**DOI:** 10.1186/s40510-019-0280-2

**Published:** 2019-06-18

**Authors:** Kaifeng Yin, Eugene Han, Jing Guo, Toshihiko Yasumura, Dan Grauer, Glenn Sameshima

**Affiliations:** 1 Department of Orthodontics, Herman Ostrow School of Dentistry at University of Southern California, 925 W 34th St, Los Angeles, CA 90089 USA; 2 Center for Craniofacial Molecular Biology, Herman Ostrow School of Dentistry at University of Southern California, Los Angeles, CA 90033 USA; 3 Corporate Practice, Houston, TX 77069 USA; 4 Department of Orthodontics, Tokyo Dental College, Tokyo, 101-0061 Japan

**Keywords:** Class II malocclusion, Carriere Distalizer, Forsus, Class II elastics, Retrospective study

## Abstract

**Background:**

The purpose of this study was to evaluate the treatment effectiveness of Carriere Distalizer in comparison to Class II intermaxillary elastics and Forsus.

**Methods:**

Three groups of patients treated with Class II intermaxillary elastics (*n* = 18), Carriere Distalizer (*n* = 18), and Forsus appliance (*n* = 18) were collected from three private orthodontic practices. Inclusion criteria were as follows: (1) 10–14 years old of start age with permanent dentition, (2) no history of previous orthodontic treatment, (3) complete pre- and post-treatment records, (4) dental Class II division 1 (end-to-end or more), (5) no pre-treatment transverse discrepancy, (6) non-extraction treatment plan, and (7) Class I post-treatment occlusal relationship. The data consisted of cephalometric and study model measurements from pre- and post-treatment records and treatment time. Two-tail Student *t* test was used to analyze the differences in cephalometric changes and dental corrections between Carriere Distalizer group and Class II elastics/Forsus group.

**Results:**

All three groups of patients showed no differences in the age of treatment initiation, pre-treatment cephalometric measurements and discrepancy index (DI). The time of Class II correction for Carriere Distalizer was significantly shorter than that for Class II elastics; there was no difference in the length of Class II correction between Carriere Distalizer and Forsus groups. The amount of Class II correction (canine/molar relationship) was significantly lower for Carriere Distalizer when compared with Forsus appliance. Carriere Distalizer, similarly to Class II elastics, did not induce any statistically significant correction in skeletal component (ANB and Wits appraisal).

**Conclusions:**

There is no clinically significant skeletal correction induced by Carriere Distalizer in growing patients. Carriere Distalizer can be applied to treatment of mild to moderate Class II dental malocclusion over 6 months on average, although the total treatment time may be prolonged due to various side effects. Overall, the Carriere Distalizer appears to be no more effective or efficient than alternatives in the treatment of Class II malocclusion.

## Background

Treatment of Class II malocclusion is a common challenge that orthodontists encounter on a daily basis. Specifically, Class II division I malocclusion caused by mandibular deficiency and/or maxillary excess is characterized by distally positioned lower molars relative to the upper molars, and protrusive appearance of upper incisors [1]. Patients with Class II division 1 often exhibit convex facial profile, recessed chin, everted lower lip, and short chin-to-neck length. These dental and soft tissue features can pose negative influences on affected children both functionally and emotionally. The prevalence of Class II division 1 malocclusion varies from 8.6% to 33.7% in the US population [2, 3]. Many different treatment modalities exist for Class II malocclusion depending on whether the problem is skeletal or dentoalveolar.

For the skeletal correction of Class II malocclusion with functional appliances (for instance, Twin Block appliance and Forsus appliance), the aim is to stimulate mandibular growth and to position the mandible forward; the appliances can be either fixed or removable [4–12]. Headgear is another conventional appliance for treatment of Class II growing patients. The rationale of headgear is to hold the maxillary growth and to allow the mandibular growth to catch up [13]. According to the most recent scientific evidence, the optimal time of applying functional appliances is during or slightly after the onset of puberty peak in growth velocity [5, 14, 15]. Treatment in late teens involves full fixed appliances with intermaxillary elastics, functional appliances (Herbst or Forsus appliance), or molar distalization mechanics supported by temporary anchorage devices (TADs). Differential extraction patterns between upper and lower arches including extraction in the upper arch, and orthognathic surgery are some alternative strategies that are frequently adopted by the practitioners. Selection of an appropriate treatment modality depends on the severity of skeletal or dental malocclusion, patients’ esthetics, and patient compliance.

**Fig. 1:**
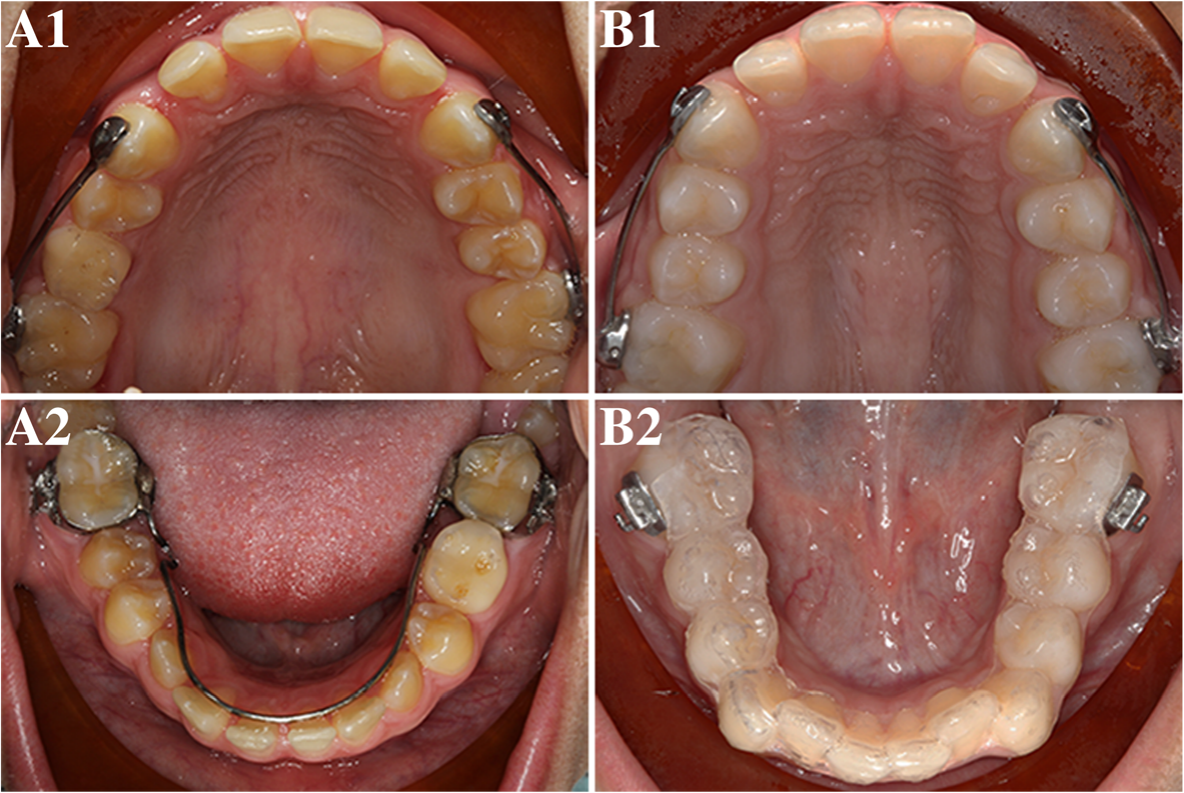
Carriere Distalizer and appliance used for the lower arch*.* Carriere Distalizer functions to distalize the posterior segment as a unit (A1 and B1). The lower arch, either banded with a lower lingual holding arch (A2) or held together with a clear retainer (B2), serves as the main anchorage source for Class II correction. Depending on the length of Carriere Distalizer, elastics can be worn from the upper canines or premolars to the lower molars

Various types of molar distalization appliances are currently available to correct dental Class II malocclusion, such as the distal jet and the pendulum device [16–20]. Carriere Motion 3D Class II appliance or Carriere Distalizer (Henry Schein Inc., New York, NY) has been marketed as a Class II corrector that functions to rotate and upright the maxillary first molars while distalizing the posterior segments as a unit [21]. The lower arch, either banded with a lower lingual holding arch or held together with a clear retainer, serves as the main anchorage source for Class II correction (Fig. 1). Depending on the span of Carriere Distalizer, elastics can be worn from the upper canines or premolars to the lower molars; these are similar to Class II intermaxillary elastics. Due to the fact that application of Carriere Distalizer usually precedes the delivery of full edgewise appliance, adolescent patient’s comfort and overall experience can be improved [22]. Although Carriere Distalizer has been growing in popularity among clinical practitioners over the last decade, few studies are available to evaluate the treatment efficiency of Carriere Distalizer for Class II correction.

## Methods

### Subjects

The study was approved by the ethics board committee of University of Southern California (IRB approval ID: UP-18-00467). Treatment records of 78 patients, treated with bilateral Class II intermaxillary elastics, Carriere Distalizer, and Forsus appliance (3 M Unitek Corp., St. Paul, MN) coupled with full edgewise appliances (MBT prescription 022″ slot, Opal Orthodontics, South Jordan, UT), were collected from three private orthodontic practices in southern California. In Class II elastics and Carriere Distalizer groups, intermaxillary elastics were applied from upper canines to lower first molars. Forsus springs extended from upper first molars to distal of lower canines’ brackets. Patient compliance was not reported as an issue of concern by the case providers. Orthodontic informed consent was obtained from all patients/parents. The inclusion criteria were (1) 10–14 years old of start age with permanent dentition, (2) no history of previous orthodontic treatment, (3) complete pre- and post-treatment records (cephalometric X-ray and digital study models), (4) dental Class II division 1 (end-to-end or more), (5) no pre-treatment transverse discrepancy, (6) non-extraction treatment plan, and (7) Class I post-treatment occlusal relationship. The 78 patients were allocated into the three corresponding groups: (1) Class II intermaxillary elastics, (2) Carriere Distalizer, and (3) Forsus appliance.

**Fig. 2:**
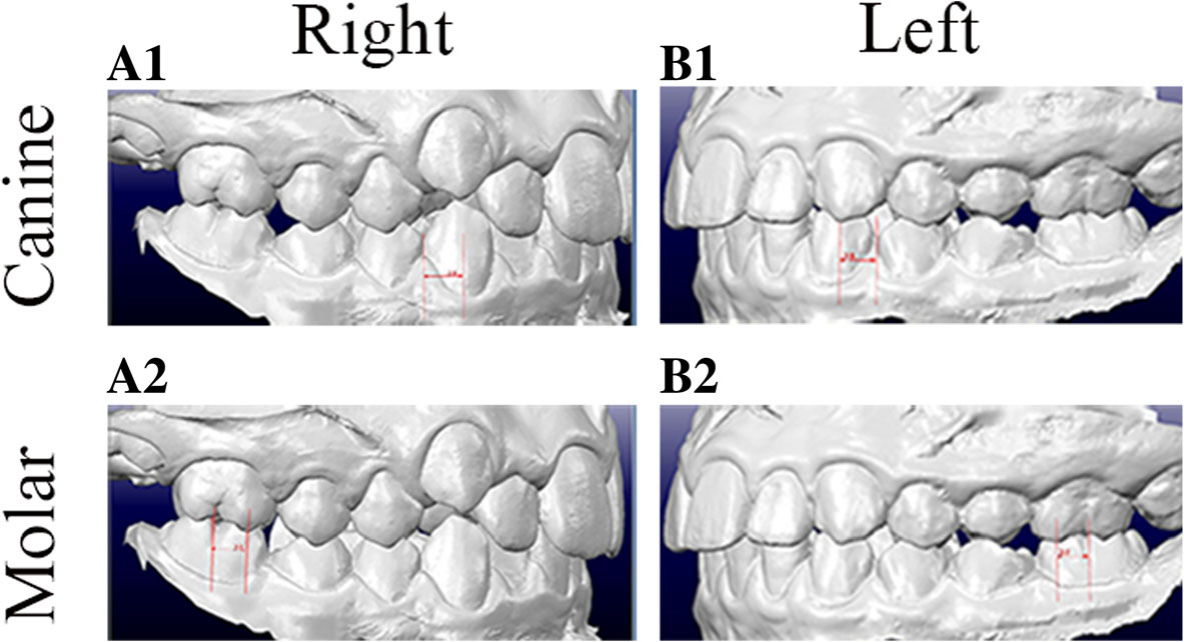
Measurement of canine and molar relationship on digital study models*.* The reference surfaces for canine relationship were the interproximal contact point between mandibular canine and first premolar and the cusp of maxillary canine (A1 and B1); the reference surfaces for molar relationship measurement were the buccal groove of mandibular first molar and the mesiobuccal cusp of maxillary first molar (A2 and B2); The distances (millimeter) between the two reference surfaces of molar and canine were recorded separately for left and right sides in each subject

A pilot study was conducted based on the records of 12 patients randomly selected from each treatment group, for the purpose of obtaining an estimate of sample size. Among all the variables tested, molar correction (Fig. 2) generated the least clinically important difference (in absolute value) between the Carriere Distalizer group and the Class II elastics/Forsus group. As a result, sample size calculation was performed using the between-group mean differences and standard deviation (SD) of molar correction, with other assumptions as follows: continuous variable, normal distribution, two independent samples—Carriere Distalizer group and Class II elastics/Forsus group, and type I error rate of 0.05 and power of 0.8 [23]. The minimum required sample size was 16. Eighteen subjects were finally assigned to each treatment group with within-group genders being balanced.

### Data collection

The baseline information of the 54 patients, including gender, age (pre-treatment), length of treatment (months), and length of Class II correction (months), was recorded from Dolphin management software (Patterson Dental Supply, Inc., Chatsworth, CA) (Table 1). The Discrepancy Index (DI) of each patient was assessed using the ABO worksheet (https://www.americanboardortho.com/media/1186/discrepancy-index-worksheet-for-print.pdf). Pre- and post-treatment cephalometric X-rays were traced on Dolphin Imaging software (Patterson Dental Supply, Inc., Chatsworth, CA). The main body of clinical variables of cephalometric analysis was extracted from multiple other analyses to represent the skeletal and dentoalveolar components in sagittal and vertical dimensions (Tables 2 and 3) [24–29]. Changes in Cephalometric measurements were calculated as the differences between post- and pre-treatment values (Table 4).

**Table 1:** Baseline information of subjects in each group. **P < 0.01

	Number of subjects (*n*)	Age (years)		Length of treatment (months)		Length of Class II correction (months)		Discrepancy index (DI)		
		Mean	SD	Mean	SD	Mean	SD	Mean	Min	Max
Class II elastics	18	12.6	1.2	23.9**	5.8	10.3**	3.9	13	7	26
Carriere Distalizer	18	11.9	1.0	32.3	8.4	6.3	2.2	12	6	22
Forsus	18	12.8	1.2	28.6	5.3	7.2	2.7	15	7	22

**Table 2:** Pre-treatment cephalometric measurements and canine/molar relationship

Pre-treatment	Class II elastics		Carriere Distalizer		Forsus	
	Mean	SD	Mean	SD	Mean	SD
Maxillary skeletal						
SNA (°)	79.9	3.6	80.4	3.1	82.0	3.0
Co-ANS (mm)	92.1	7.0	89.5	6.9	94.1	9.0
Mandibular skeletal						
SNB (°)	75.4	2.9	76.1	2.8	76.7	2.3
Co-Gn (mm)	116.2	8.1	117.5	10.2	119.3	11.0
Maxillary/mandibular						
ANB (°)	4.5	2.2	4.2	2.0	5.3	2.4
Wits (mm)	3.2	1.9	2.6	2.3	4.5	2.8
Maxillary dental						
U1-SN (°)	98.0	7.8	100.5	7.6	102.6	7.7
U1-NA (°)	18.1	9.0	20.1	6.3	20.7	8.7
U1-NA (mm)	2.4	3.2	3.5	2.4	2.9	4.0
Mandibular dental						
IMPA (°)	95.1	8.3	94.0	7.9	95.3	6.9
L1-NB (°)	22.8	8.2	23.6	6.4	23.0	5.5
L1-NB (mm)	3.9	3.0	4.6	2.3	4.6	1.9
Interdental						
Interincisal angle (°)	131.8	12.1	132.0	9.9	131.1	9.1
Overjet (mm)	5.1	1.3	4.8	0.8	6.3	2.6
Overbite (mm)	4.2	2.9	4.0	1.4	5.0	2.0
Vertical						
Upper facial height (mm)	54.3	6.1	49.5	3.2	53.6	5.0
Lower facial height (mm)	64.1	6.8	58.5	5.2	69.0	6.8
FMA (°)	25.1	5.0	24.7	5.6	23.9	4.1
Sn-GoGn (°)	29.5	4.8	30.8	5.2	29.6	3.5
Canine relationship (mm)	4.3	1.2	3.4	1.8	5.2	2.5
Molar relationship (mm)	4.2	1.2	4.3	1.3	4.5	1.2

**Table 3:** Post-treatment cephalometric measurements and canine/molar relationship

Post-treatment	Class II elastics		Carriere Distalizer		Forsus	
	Mean	SD	Mean	SD	Mean	SD
Maxillary skeletal						
SNA (°)	79.8	3.8	80.9	4.4	80.7	3.1
Co-ANS (mm)	93.7	4.3	90.8	5.6	95.9	8.0
Mandibular skeletal						
SNB (°)	76.3	3.4	77.3	3.6	78.3	2.8
Co-Gn (mm)	121.3	5.6	124.2	7.0	126.8	12.2
Maxillary/mandibular						
ANB (°)	3.5	1.7	3.7	2.2	2.4	2.1
Wits (mm)	1.7	1.3	1.2	1.2	0.3	2.8
Maxillary dental						
U1-SN (°)	106.7	5.2	106.2	6.0	108.0	4.3
U1-NA (°)	26.9	5.0	25.3	4.8	27.2	5.4
U1-NA (mm)	4.4	1.5	6.5	2.9	5.5	2.1
Mandibular dental						
IMPA (°)	101.6	5.5	98.7	7.6	99.8	6.0
L1-NB (°)	28.9	5.2	30.0	7.5	27.5	5.3
L1-NB (mm)	6.7	2.5	6.8	2.3	6.4	2.1
Interdental						
Interincisal angle (°)	120.6	7.8	120.9	9.9	122.8	6.5
Overjet (mm)	2.9	0.7	2.8	0.5	3.0	1.0
Overbite (mm)	2.6	0.6	2.2	0.8	2.7	1.0
Vertical						
Upper facial height (mm)	55.1	5.7	51.3	2.8	54.5	5.7
Lower facial height (mm)	68.6	4.8	64.8	4.0	72.6	6.7
FMA (°)	24.6	5.7	26.4	6.8	22.2	4.5
Sn-GoGn (°)	27.9	4.9	30.8	6.6	26.2	4.2
Canine relationship (mm)	0.8	1.0	0.6	0.8	0.8	0.9
Molar relationship (mm)	−0.25	0.9	−0.8	1.0	−0.6	0.9

**Table 4:** Pre- and post-treatment changes in cephalometric measurements and canine/molar relationship correction

Post-Pre treatment	Class II elastics		Carriere Distalizer		Forsus	
	Mean	SD	Mean	SD	Mean	SD
Maxillary skeletal						
SNA (°)	− 0.1	2.1	0.6	2.4	−1.2	2.5
Co-ANS (mm)	1.6	5.2	1.3	4.4	1.8	6.0
Mandibular skeletal						
SNB (°)	0.95	1.5	1.2	1.9	1.6	1.5
Co-Gn (mm)	5.1	4.6	6.7	6.8	7.5	6.6
Maxillary/mandibular						
ANB (°)	−1.0	1.6	−0.5	1.5	−2.9^***^	1.7
Wits (mm)	−1.5	2.0	−0.5	2.3	−4.2^***^	2.7
Maxillary dental						
U1-SN (°)	8.7	10.2	5.7	7.7	5.4	8.6
U1-NA (°)	8.8	11.3	5.1	7.1	6.5	9.4
U1-NA (mm)	2.0	2.7	3.0	7.7	2.5	3.7
Mandibular dental						
IMPA (°)	6.4	7.4	4.7	7.9	4.5	5.7
L1-NB (°)	6.1	6.5	6.4	6.4	4.5	5.9
L1-NB (mm)	2.8	1.4	2.2	1.9	1.8	1.8
Interdental						
Interincisal angle (°)	−11.2	15.6	−11.1	11.1	−8.3	11.2
Overjet (mm)	−2.2	1.3	−2.0	0.9	−3.2	2.4
Overbite (mm)	−2.6	2.8	−1.8	1.2	−3.2	2.2
Vertical						
Upper facial height (mm)	0.85	2.2	1.9	4.0	0.9	3.3
Lower facial height (mm)	4.5	4.1	6.3	2.6	3.5	3.3
FMA (°)	−0.48	3.8	1.7	4.8	−1.7	4.7
Sn-GoGn (°)	−1.6	2.6	−0.1	4.1	−1.4	1.9
Canine correction (mm)	−3.8	1.4	−3.7	1.7	−5.2^**^	2.3
Molar correction (mm)	−3.5	1.5	−3.5	1.7	−4.5^*^	2.2

*P < 0.05, **P < 0.01, ***P < 0.001

In order to evaluate the amount of Class II correction, pre-treatment molar and canine relationships were first measured using digital study models on centric occlusion (CO) supported by OrthoCAD software (Align Technology, Inc., San Jose, CA) (Fig. 2). The reference surfaces for molar relationship measurement were the buccal groove of mandibular first molar and the mesiobuccal cusp of maxillary first molar; the reference surfaces for canine relationship were the interproximal contact point between mandibular canine and first premolar and the cusp of maxillary canine. The distances (millimeter) between the two reference surfaces of molar and canine were recorded separately for left and right sides in each subject. Although all the patients were finished with Class I molar and canine relationship, the distances between reference surfaces from post-treatment study models were measured again. The differences between pre- and post-treatment measurements were then calculated to obtain the relative amount of Class II correction of molar and canine (Table 4).

### Statistical analysis

Data collection procedures were carried out by two researchers independently and repeated at an interval of 6 weeks. For the dataset of each clinical variable evaluated, the assumption of normality distribution was not violated (Shapiro-Wilk test, *α* = 0.05) (IBM SPSS Statistics, IBM Corporation, NY). The intraclass coefficients (ICC) calculated for all the clinical variables showed excellent inter-rater and intra-rater reliability of measurements—ICC > 0.75 (data not shown, IBM SPSS Statistics). Therefore, an average was taken from the multiple measurements of each clinical variable. Finally, two-tail Student’s *t* test was applied to detect the potential differences in start age, DI, length of treatment, length of Class II correction, cephalometric changes, and Class II correction—between Carriere Distalizer group and Class II elastics/Forsus group (*α* = 0.05).

## Results

### Baseline information

The pre-treatment age of subjects in Carriere Distalizer group was 11.9 ± 1.0 (mean ± sd), while that of subjects in Class II elastics and Forsus groups were 12.6 ± 1.2 and 12.8 ± 1.2, respectively (Table 1). The differences in the start age between Carriere Distalizer and Class II elastics group and between Carriere Distalizer and Forsus group were not statistically significant. The ABO DI scores of the samples in three groups showed similar statistical dispersions, which ranged from 6 to 26 (Table 1). The mean DI scores showed no statistical differences between Carriere Distalizer group and Class II elastics/Forsus group. All other clinical characteristics as measured by cephalometric analysis were also statistically comparable between the treatment groups (Table 2).

### Total treatment time and time for elastics/appliance use

**Fig. 3:**
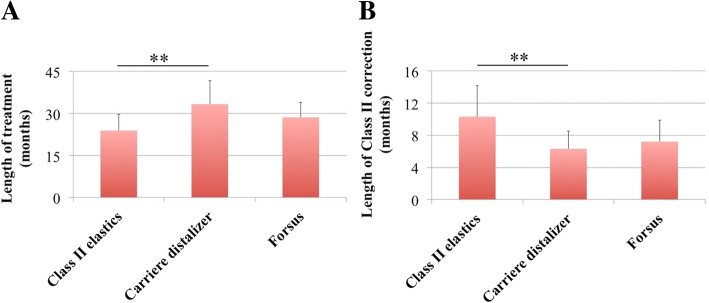
**a**, **b** Length of treatment (total treatment time) and length of Class II correction (time for elastics/appliance use). *P < 0.05, **P < 0.01, ***P < 0.001

Based on the subjects collected for the study, the length of treatment (total treatment time) with Carriere Distalizer as the Class II corrector was 32.3 ± 8.4 months (Table 1). For Forsus group, the length of treatment was 28.6 ± 5.3 months, which was not statistically different from that of Carriere Distalizer group. The length of treatment with conventional Class II elastics was 23.9 ± 5.8 months—significantly shorter when compared with Carriere Distalizer group (*P* = 0.002) (Table 1 and Fig. 3). However, the length of Class II correction (time for elastics/appliance use) for Carriere Distalizer (6.3 ± 2.2 months) was significantly shorter than that for Class II elastics (10.3 ± 3.9 months) (*P* = 0.005), while there was no statistical difference between Carriere Distalizer and Forsus (7.2 ± 2.7 months) groups (Table 1 and Fig. 3).

### Quantification of Class II correction

**Fig. 4:**
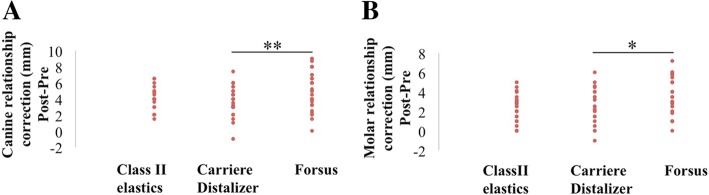
**a**, **b** Quantification of Class II correction based on measurements from pre- and post-treatment study models. *P < 0.05, **P < 0.01, ***P < 0.001

The amount of Class II corrections as calculated from pre- and post-treatment study models is presented in the main text as absolute values for a better understanding. Class II molar relationship correction was significantly lower for Carriere Distalizer (3.7 ± 1.7 mm) when compared to that of Forsus appliance (5.2 ± 2.3 mm) (*P* = 0.046, Table 4 and Fig. 4a). The mean difference in canine relationship correction was highly significant between Carriere Distalizer (3.5 ± 1.7 mm) and Forsus groups (4.5 ± 2.2 mm) (*P* = 0.009, Table 4 and Fig. 4a). In contrast, the Class II treatment mechanics involving Carriere Distalizer and intermaxillary elastics showed no statistically significant difference in correction of both molar and canine relationships (Table 4 and Fig. 4b).

### Cephalometric changes

**Fig. 5:**
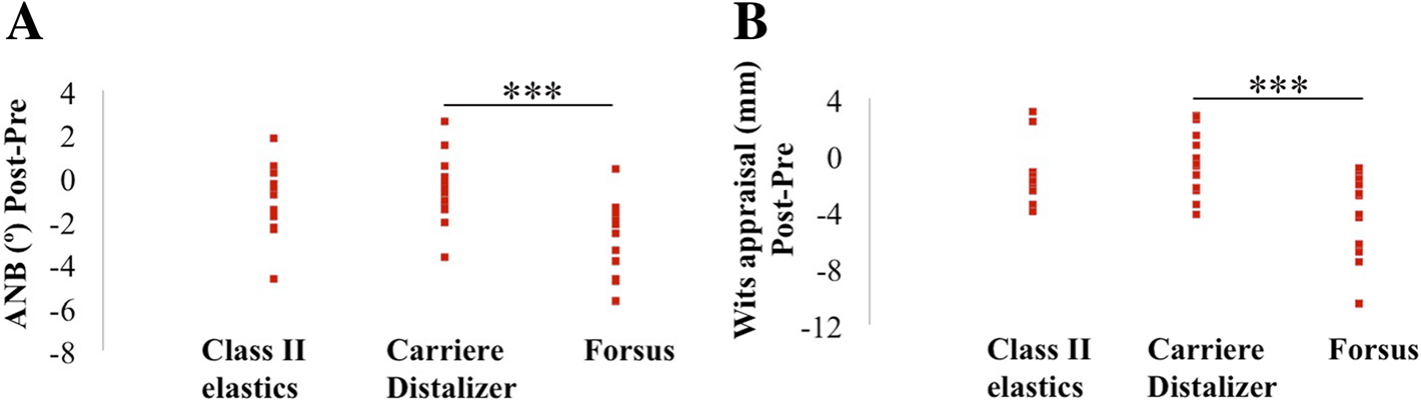
**a**, **b** Pre- and post-treatment differences in ANB and Wits appraisal. *P < 0.05, **P < 0.01, ***P < 0.001

**Fig. 6:**
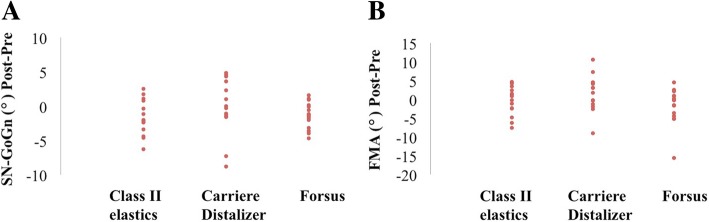
**a**, **b** Pre- and post-treatment changes in SN-GoGn and FMA. *P < 0.05, **P < 0.01, ***P < 0.001

**Fig. 7:**
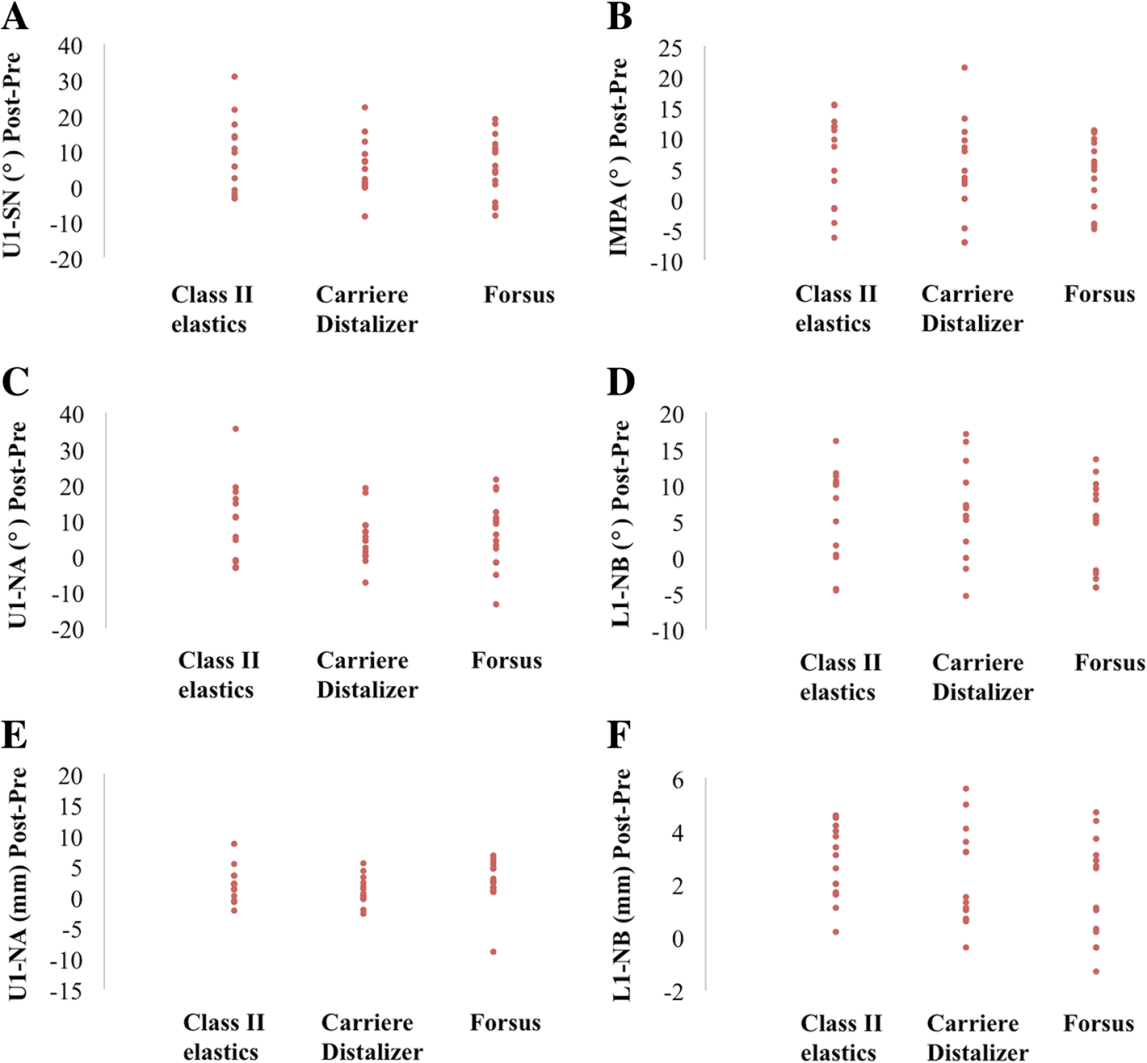
**a**–**f** Pre- and post-treatment changes in maxillary and mandibular dental measurements. *P < 0.05, **P < 0.01, ***P < 0.001

Pre- and post-treatment cephalometric measurements are presented in Tables 2 and 3. For the Carriere Distalizer group, pre- and post-treatment differences in ANB and Wits appraisal were both statistically similar to those in Class II elastics group. However, Forsus group showed a significantly higher amount of changes in ANB and Wits than Carriere Distalizer group (ANB *P* = 0.001; Wits *P* = 0.001) (Table 4 and Fig. 5). Except for ANB and Wits, all the other cephalometric variables showed no statistically significant differences in pre- and post-treatment changes (Table 4, Figs. 6 and 7).

## Discussion

To evaluate the efficiency of using Carriere Distalizer to treat Class II division I non-extraction patients, we compared the length of treatment, the length of Class II correction, the amount of Class II correction and pre- and post-treatment differences in cephalometric measurements between growing patients treated by Carriere Distalizer and conventional Class II intermaxillary elastics or Forsus appliance. Patient data collected for the study showed no differences in baseline information (Tables 1, 2).

**Fig. 8:**
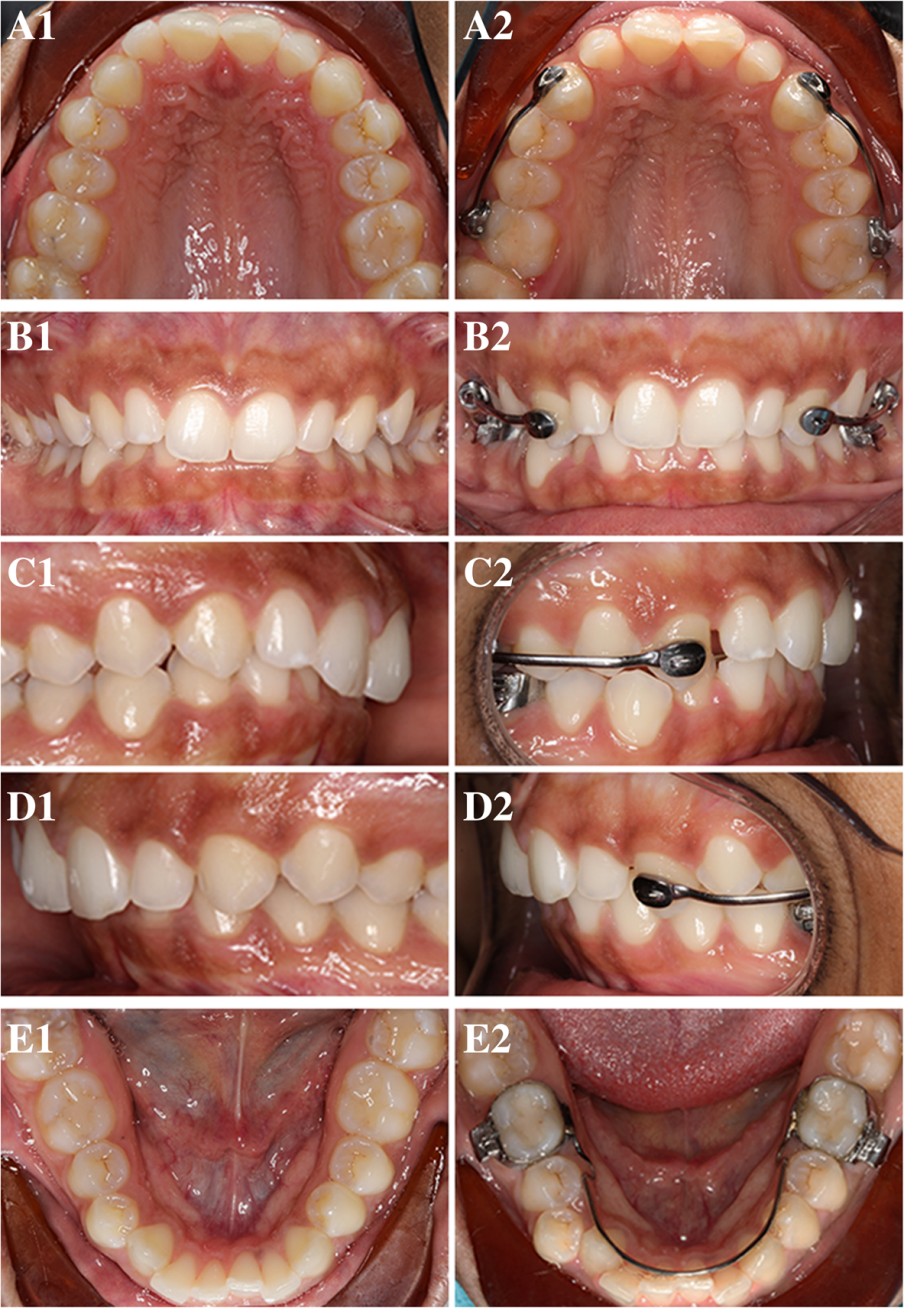
Potential side effects induced by Carriere Distalizer*.* A1, B1, C1, D1, and E1 pre-treatment intraoral photos. A2, B2, C2, D2, and E2 intraoral photos taken after 4 months of treatment with Carriere Distalizer. The intermaxillary elastics supported by Carriere Distalizer and lower lingual holding arch or clear retainer may result in extra space distal to maxillary lateral incisors (A2, C2, and D2), extrusion of maxillary canines (B2, C2, and D2), and worsening in arch length discrepancy in the mandibular arch (E2-lower right buccal segment)

**Fig. 9:**
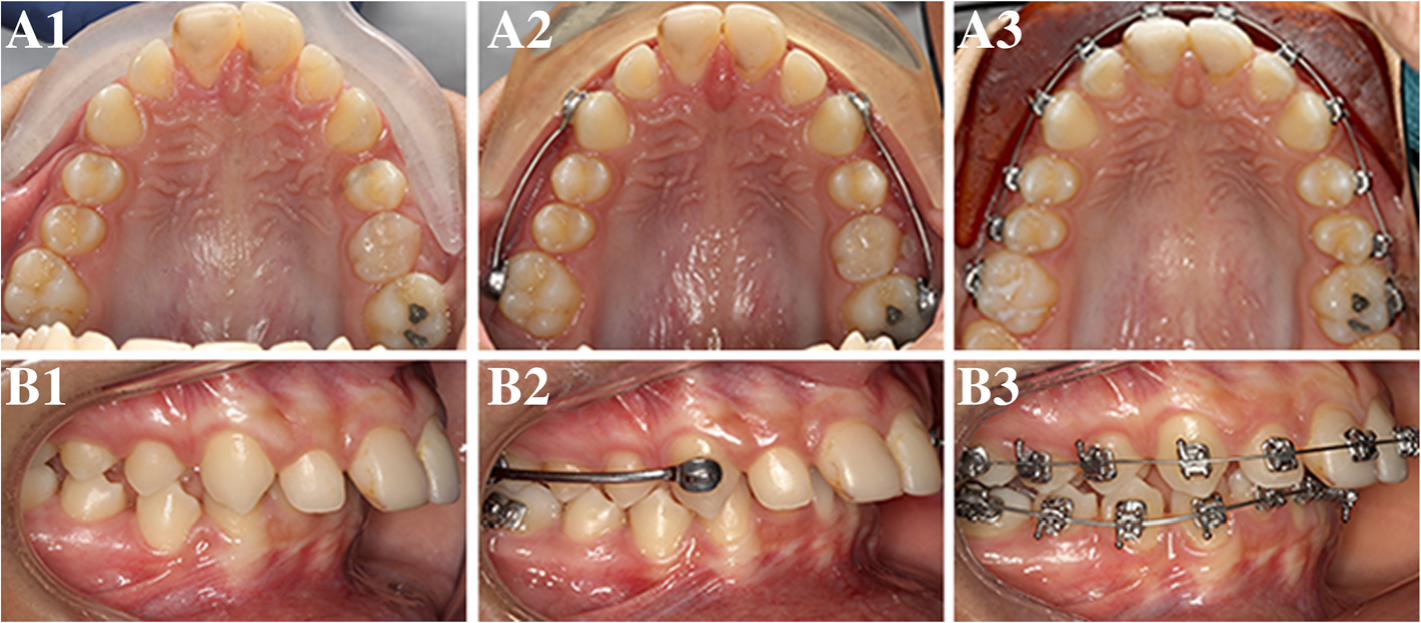
Relapse of derotated molar with Carriere Distalizer*.* A1 and B1 pre-treatment intraoral photos. A2 and B2 progress photos after 3 months of treatment with Carriere Distalizer. A3 and B3 progress photos 1 month after removal of Carriere Distalizer. The right molar relationship was corrected to Class I with Carriere Distalizer (B2) and part of the correction was achieved by derotating the upper right first molar (A2). However, there was a relapse of derotated upper right first molar after Carriere Distalizer was removed (B3)

In terms of treatment time, the potential difference in the length of treatment was not statistically significant between Carriere Distalizer and Forsus groups (Fig. 3a). According to the statistics collected from the current patient samples, treatment with either of these two Class II correction appliances requires an average of approximately 2.5 years (Table 1). By comparison, the average length of treatment with intermaxillary elastics as the Class II correction mechanics was 5–6 months shorter than that with Carriere Distalizer (Fig. 3a). Class II correction with Carriere Distalizer usually precedes the delivery of full edgewise appliance. Unlike the conventional Class II elastics supported by upper and lower edgewise appliance and full-size wires, the intermaxillary elastics supported by Carriere Distalizer and lower lingual holding arch or clear retainer may result in extra space distal to maxillary lateral incisors, extrusion of maxillary canines, and worsening in arch length discrepancy in the mandibular arch (Fig. 8). Additionally, due to the existence of a ball-and-socket joint on top of the pad bonded to maxillary first molars, part of the Class II correction induced by Carriere Distalizer is achieved by derotating the maxillary first molars distally (Fig. 9). Without an active retention device such as a transpalatal arch, relapse of derotated molars is unavoidable and needs to be re-corrected into the treatment [30, 31]. Therefore, although sagittal discrepancy in the buccal segments may not be a concern in the later stage of the treatment, these factors mentioned above could potentially increase the complexity of the treatment after Carriere Distalizer is removed, resulting in prolonged total treatment time.

The average length of Class II correction or appliance/elastics use for Carriere Distalizer group was comparable to that of Forsus group (Fig. 3b). An average of 6–7 months is required for Carriere Distalizer to correct an end-to-end sagittal discrepancy (Tables 1 and 2). Considering the significantly higher amount of correction in molar and canine relationships and the potential skeletal correction induced by Forsus, Class II correction with Carriere Distalizer in growing patients is not as efficient as with Forsus appliance (Figs. 3 and 4). However, the efficiency of Carriere Distalizer in correcting posterior sagittal discrepancy is still superior to that of Class II intermaxillary elastics; for similar amount of Class II correction, Class II elastics requires 4 months more on average compared to Carriere Distalizer (Table 1 and Fig. 3b).

In our study, we calculated the differences in canine/molar relationship between pre- and post-treatment digital study models, as the amount of Class II correction. Thus, the measurements in canine and molar relationship correction are combinations of both dentoalveolar and skeletal correction generated by the utilization of Carriere Distalizer and the control appliances/mechanics. With Carriere Distalizer, an average of 3.5–3.7 mm of canine and molar correction was obtained, which is similar to the amount of Class II correction with Class II elastics (Table 4 and Fig. 3). Forsus appliance was more effective than Carriere Distalizer in the correction of sagittal discrepancy; the Class II correction achieved with Forsus appliance was an averaged of 4.5–5.2 mm, approximately 1 mm more than that with Carriere Distalizer (Table 4). All patients collected for the study were finished with Class I canine and molar relationship, which are considered clinically acceptable treatment results. However, when the post-treatment study models were measured using the methodology mentioned in the “Methods” section, most cases did not show a zero distance between reference surfaces defined for canine/molar relationship. Therefore, although statistically significant differences were detected in Class II correction between Carriere Distalizer and Forsus groups, the differences may not be clinically significant.

In order to better understand the difference in Class II correction, comparison of cephalometric changes reflecting skeletal components were performed between treatment groups. ANB and Wits appraisal were the only clinical variables that showed statistical significance: pre- and post- treatment changes in Carriere Distalizer group were significantly lower than those in Forsus group but were not statistically different from those of Class II elastics group (Table 4 and Fig. 5). Based on the scientific evidences currently available, the treatment effect of Class II elastics is primarily dentoalveolar, while functional appliance like Forsus or Herbst appliance may impact the growth of maxilla and mandible in growing patients in spite of the potential controversies [12, 32, 33]. As a result, the lower amount of correction associated with the application of Carriere Distalizer is attributed to only dentoalveolar correction in the sagittal dimension; the correction of a mild-to-moderate dental Class II can be realized with Carriere Distalizer. Finally, the potential between-group differences in dental and vertical cephalometric changes were not statistically significant, which indicates that desirable orthodontic dentoalveolar corrections can be equally achieved with Carriere Distalizer followed by full edgewise appliance (Figs. 6 and 7).

Like most orthodontic appliances, the success of Carriere Distalizer depends on patient compliance with intermaxillary elastics and lower retainer wear when Carriere Distalizer is in place. A series of other patient-related factors may also need to be evaluated before commencing the treatment with Carriere Distalizer, such as vertical skeletal pattern, torque of lower incisors, and inclination of occlusal plane. Our group recently treated a 14-year-old male patient with Carriere Distalizer coupled with a lower clear retainer for 8 months but failed to obtain any treatment effects as expected from Carriere Distalizer. According to the superimposition of the cephalometric radiographs taken before appliance delivery and immediately after appliance removal, maxillary arch was barely distalized with anchorage loss in the lower arch. While patient compliance was ruled out as the culprit because of anchorage loss in the lower arch and the fit of lower clear retainer, a low-angle skeletal pattern (vertically) was suspected as the major factor that resulted in the failure of treatment. Therefore, for the purpose of achieving better clinical outcomes with Carriere Distalizer, a further investigation regarding the potential failure factors of applying Carriere Distalizer to treat Class II patients is warranted.

## Conclusions

There is no clinically significant skeletal correction induced by Carriere Distalizer in growing patients. Carriere Distalizer can be applied to treatment of mild to moderate Class II dental malocclusion over 6 months on average, although the total treatment time may be prolonged due to various side effects. Taken together, it is not effective and efficient to treat Class II malocclusions using Carriere Distalizer.

## Data Availability

All data generated and analyzed during this study were included in this manuscript.

## Abbreviations

CO: Centric occlusion
DI: Discrepancy index
ICC: Intraclass coefficient
SD: Standard deviation
TADs: Temporary anchorage devices
